# Retroperitoneal dedifferentiated liposarcoma with colon infiltration: a case report and literature review

**DOI:** 10.3389/fonc.2025.1724071

**Published:** 2025-12-10

**Authors:** Xi Tu, Xiyao Zhuang, Chaoyou Huang

**Affiliations:** 1 Department of Urology, West China School of Medicine, Sichuan University, Sichuan University Affiliated Chengdu Second People’s Hospital, Chengdu Second People’s Hospital, Chengdu, Sichuan, China; 2 Department of Internal Medicine, Chengdu Shuangliu Hospital of Traditional Chinese Medicine, Chengdu, Sichuan, China

**Keywords:** colonic invasion, dedifferentiated tumor, liposarcoma, oncological surgery, retroperitoneal neoplasia, soft tissue sarcoma

## Abstract

Retroperitoneal dedifferentiated liposarcoma (RP DDLS) is considered highly malignant, with a high recurrence rate and poor prognosis, and is usually asymptomatic until the advanced stages. Retroperitoneal liposarcoma with colon invasion is extremely rare, with only a few cases reported in the literature. This study reported the case of a 75-year-old male patient with a retroperitoneal space-occupying lesion without any clinical symptoms. Abdominal computed tomography (CT) and magnetic resonance imaging (MRI) scans suggested a neoplastic lesion. After adequate preoperative preparation, the patient underwent laparoscopic tumor resection and right hemicolectomy. Pathological and immunohistochemical examinations of the resected retroperitoneal mass and right hemicolon revealed dedifferentiated liposarcoma (DDLPS). Dedifferentiated liposarcomas are characterized by local invasiveness and a high recurrence rate. The association between dedifferentiated liposarcoma and invasive behavior observed in this patient underscores the importance of multidisciplinary collaboration as the primary treatment strategy. Nevertheless, complete tumor resection remains crucial for improving the prognosis and reducing the high local recurrence rate.

## Introduction

Retroperitoneal liposarcoma is a rare and complex tumor originating from mesenchymal tissue, accounting for approximately 0.07% to 0.2% of all malignant tumors and approximately 15% of soft tissue sarcomas (1). These masses are usually detected incidentally or present at an advanced stage owing to their anatomical location and slow growth. Despite their large size, histological invasion of adjacent organs is uncommon; they typically exhibit compressive rather than infiltrative behavior (2). Retroperitoneal liposarcomas are considered highly malignant and have a high recurrence rate, with a 5-year survival rate of approximately 42.6% for dedifferentiated tumors (3). CT and ultrasound-guided aspiration biopsy are helpful for the preoperative diagnosis and evaluation of patients. Surgical resection of negative margins is the preferred treatment option. Retroperitoneal liposarcoma with colonic infiltration is extremely rare. Herein, we present a case of a rare dedifferentiated retroperitoneal liposarcoma with colon infiltration, which is helpful for clinicians to further understand the clinical characteristics, treatment strategies of dedifferentiated liposarcoma, and the mechanism of colonic invasion.

## Case presentation

**Figure 1:**
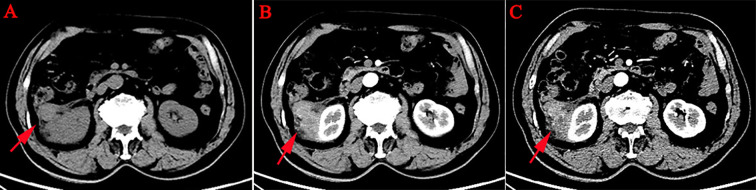
Abdominal CT. This image showed flocculent, nodular, and cord-like mixed-density lesions in the right perirenal space. The largest nodule measured approximately 3.3 cm × 2.7 cm with ill-defined borders, causing slight compression of the right kidney, and the solid components of the lesions showed enhancement. Additionally, the right lateral conical fascia and prerenal fascia were thickened, with obvious enhancement of the nodular shadows within them [**(A–C)**, red arrow].

**Figure 2:**
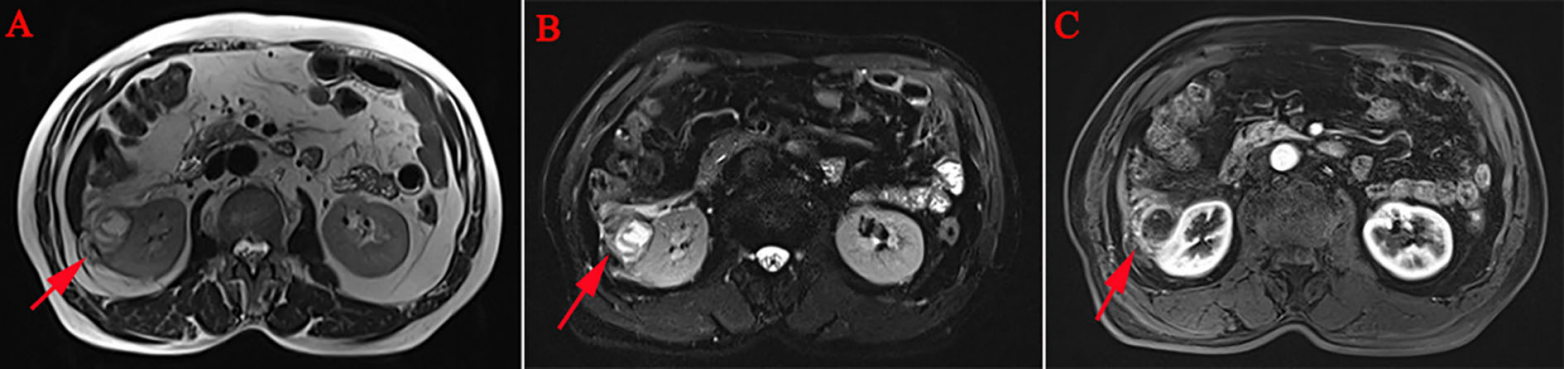
Abdominal MRI: This image showed flocculent, nodular, and cord-like mixed-signal lesions in the right perirenal space. The largest nodule measured approximately 3.0 cm × 2.4 cm with ill-defined borders, causing slight compression of the right kidney, and the solid components of the lesions exhibited enhancement. In addition, the right lateral conical fascia and prerenal fascia were thickened, with clearly enhancing nodular shadows observed within them [**(A–C)**, red arrow].

**Figure 3:**
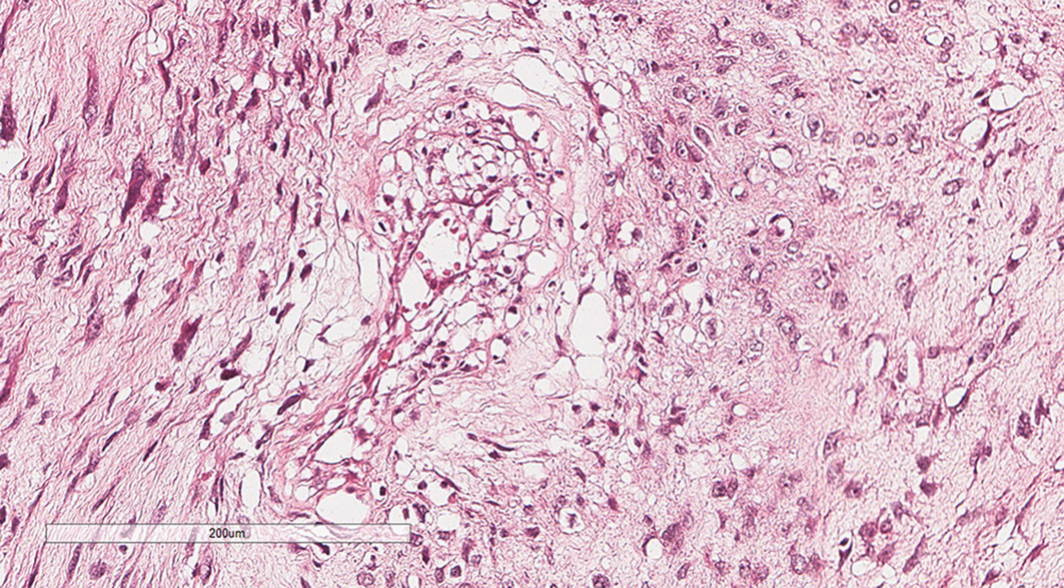
Pathology. This image showed the tumor cells were spindle-shaped and pleomorphic; In some areas, the stroma was myxoid, which contains a small amount of well-differentiated adipocytic components and heterologous differentiation components [H&E, 100x].

**Figure 4:**
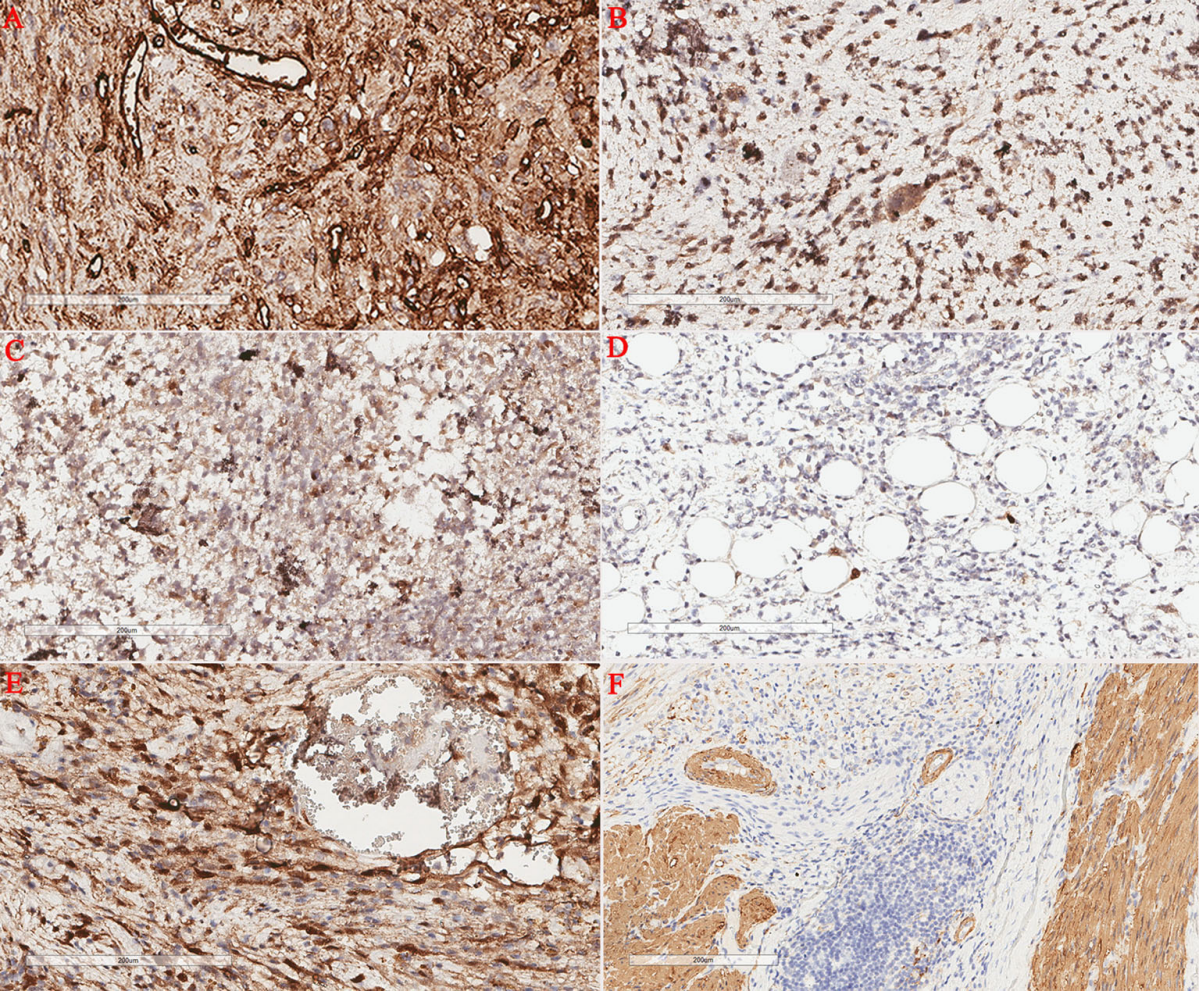
Immunohistochemical staining. Retroperitoneal tumor cells showed positive CD34 expression **(A)**; Retroperitoneal tumor cells showed positive CDK4 expression **(B)**; Retroperitoneal tumor cells showed positive P16 expression **(C)**; The *in situ* fluorescence hybridization test revealed MDM2 gene amplification **(D)**. Retroperitoneal tumor cells showed positive S100 expression **(E)**; Retroperitoneal tumor cells showed positive SMA expression **(F)**.

A 75-year-old male patient was admitted to the hospital for the detection of a retroperitoneal space-occupying lesion during physical examination. During the disease course, the patient occasionally experienced dull pain in the right waist, without abdominal distension, abdominal pain, diarrhea, hematochezia, dizziness, or palpitations. The patient had not received any specific treatment previously and had no family history of the disease or similar diseases. Three months ago, the patient underwent gastroscopy and colonoscopy due to upper abdominal pain, which revealed a neoplasm of approximately 3mm in size in the transverse colon. The patient underwent endoscopic resection of the transverse colonic lesion, and the abdominal pain improved after the operation. The pathological diagnosis of the resected lesion was hyperplastic polyp. The patient’s vital signs were normal. There was no swelling, tenderness or pain induced by tapping over either kidney area. Blood test indicators were within the normal range. Abdominal CT revealed a nodular slightly hyperdense shadow in the perirenal space on the right side, measuring approximately 3.3 cm × 2.7 cm, with ill-defined borders, compressing the right kidney. The solid component showed enhancement, and a neoplastic lesion with possible hemorrhage was suspected (**Figure 1**). Abdominal MRI revealed a neoplastic lesion (**Figure 2**). To further confirm the diagnosis and provide treatment, we planned to perform laparoscopic tumor resection. The patient was regularly administered phenoxybenzamine 10 mg p.o. tid for 1 week. After adequate preoperative preparation, the patient was scheduled to undergo laparoscopic resection of the right retroperitoneal tumor. During the operation, a retroperitoneal tumor approximately 4.0 cm × 3.0 cm in size was identified. The tumor involved the hepatic flexure of the colon, ascending colon, and mesocolon, which made dissection difficult. After obtaining consent from the patient’s family, the patient also underwent laparoscopic right hemicolectomy. Pathological examination of the resected retroperitoneal mass and right hemicolon revealed a spindle-cell tumor (**Figure 3**). Immunohistochemistry showed that the tumor cells were positive for the expression of CD34, S100, SMA, Desmin, P16, CDK4, MDM2, RB1, and MyoD1, with a Ki-67 index of 30%-40%, and negative for EMA, PCK, CD117, Dog-1, MUC4, Myogenin, and HMB45 (**Figure 4**). Immunofluorescence staining revealed MDM2 gene amplification. Pathological and immunohistochemical analyses confirmed a dedifferentiated liposarcoma. The patient was discharged after surgery. During the 12-month follow-up period after surgery, no tumor recurrence was observed. However, the patient was advised to undergo regular reexamination and lifelong follow-up.

## Discussion

Retroperitoneal liposarcomas are rare malignant tumors, accounting for 15%-20% of all soft tissue sarcomas, with an overall incidence of 0.3-0.4 per 100,000 population annually (4, 5). Although retroperitoneal liposarcomas can occur in any age group, their peak incidence is in the sixth to seventh decades of life, with no significant sex or racial predilection (6). The etiology of retroperitoneal liposarcoma remains unclear. However, the European Society for Medical Oncology (ESMO) has identified several predisposing factors for retroperitoneal liposarcoma, such as genetic abnormalities (neurofibromatosis type 1, Li-Fraumeni syndrome, FAP/Gardner syndrome), ionizing radiation exposure (especially in children who have undergone multiple computed tomography scans), and diabetes mellitus (7, 8).

Patients with retroperitoneal liposarcoma often present with symptoms such as abdominal distension, pain, nausea, and vomiting due to compression of the abdominal organs. Diagnosis and treatment are challenging because of the large tumor size, complex anatomical relationships, and abundant blood supply. Liposarcomas are classified as well-differentiated, myxoid, dedifferentiated, pleomorphic, and undifferentiated (9). The well-differentiated subtype is the most common sarcoma occurring in the retroperitoneum, with a favorable prognosis and 5-year survival rate of 90% (10). The annual incidence of dedifferentiated liposarcoma is less than 0.1 cases per million people (11), which makes our case even rarer. Dedifferentiated tumors have a high tendency for local recurrence, metastasis, and poor prognosis (12).

As a histological classification system, the Fédération Nationale des Centres de Lutte Contre le Cancer (FNCLCC) has been widely used to assess the aggressiveness of soft tissue sarcomas. This system classifies tumors into three grades (grade 1, 2, and 3) based on differentiation, mitotic index, and tumor necrosis (13). It has been reported that grade 1 and grade 2 liposarcomas receiving early intervention show significant local benefits, while grade 3 tumors are significantly associated with an increased likelihood of distant metastasis (4, 14). Tumor differentiation grade has also been identified as a key prognostic factor after surgery. The main reason for this may be attributed to the unique biological characteristics of poorly differentiated tumors. Low-grade tumor cells typically exhibit strong proliferative and invasive capacities. On the other hand, poorly differentiated tumors often show more extensive vascular and perineural invasion, which may lead to residual micrometastases that are difficult to completely eradicate during surgery (14).

Contrast-enhanced abdominal computed tomography is the preferred method for evaluating retroperitoneal liposarcoma (15). These imaging techniques not only provide detailed information about the tumor’s anatomical location, size, and adjacent organs but also help assess the relationship between the tumor and adjacent visceral and neurovascular structures, which is crucial for formulating our surgical plan. On CT, retroperitoneal dedifferentiated liposarcoma presents as soft tissue density or mixed density. Among these findings, ground-glass density foci suggest the possible presence of myxoid components, and calcification or ossification can be seen in some cases. On contrast-enhanced CT and MRI, the dedifferentiated components typically exhibit marked heterogeneous enhancement, with central cystic change and necrosis (15–17). Although needle biopsy is the gold standard for diagnosing liposarcoma, it is not recommended unless the patient is unfit for surgery due to physical condition or requires preoperative chemotherapy or radiotherapy (18). MDM2 amplification is detected in nearly all atypical lipomatous tumors (ALT), well-differentiated liposarcomas (WDL), and dedifferentiated liposarcomas, and is regarded as the driver gene of these tumors (19, 20). Therefore, fluorescence *in situ* hybridization (FISH) for detecting MDM2 gene amplification at the DNA level and immunohistochemistry for assessing MDM2 protein overexpression are effective auxiliary diagnostic tools (19–22). In particular, FISH-based detection of MDM2 gene amplification status has been universally recognized by the medical community as the “gold standard” for the diagnosis of ALT, WDL, and DDL.

Surgical resection is the preferred treatment for liposarcoma and serves as the primary prognostic factor for recurrence and survival. Complete tumor resection remains the main therapeutic goal for *de novo* tumors or recurrent tumors. However, achieving negative margins can be challenging owing to the proximity of neurovascular structures, such as the aorta, vena cava, and spine, as well as the difficulty in distinguishing between normal adipose tissue and well-differentiated tumor tissue. Nevertheless, adjacent organ involvement often occurs because of tumor growth or compression. Complete surgical resection of the tumor is the most critical part of treatment for these patients. Even if combined organ resection is required, the tumor must be resected as completely as possible, which can reduce the risk of tumor recurrence and improve the patient’s chance of survival. However, even with tumor resection achieving negative margins, it remains difficult to completely prevent recurrence (23), which may be associated with the biological behavior of dedifferentiated tumors. Retroperitoneal liposarcoma has a high recurrence rate; specifically, the recurrence rate of dedifferentiated liposarcoma is as high as 80% with progressively shortened recurrence intervals (24, 25). Most patients experience multiple recurrences and require multiple surgery procedures. Most patients die of repeated recurrences. The high recurrence rate remains a major challenge in the current treatment of retroperitoneal liposarcoma. Therefore, relying solely on surgery is insufficient to fully address the high recurrence rate of DDLPS and comprehensive treatment approaches are required to improve patient prognosis.

Adjuvant therapy has been proposed as a salvage approach in cases of surgical resection with positive margins; however, its efficacy has been limited (26). A meta-analysis by Li et al. analyzed more than 30,000 patients and compared surgical treatment with and without adjuvant therapy (radiotherapy or chemotherapy). The conclusion was that patients who received adjuvant radiotherapy had a 20% reduction in the risk of death compared to those who received surgery alone (27). However, chemotherapy did not improve survival; instead, it increased the risk of death by 11% (27). For patients with localized and resectable recurrence, repeated resection remains the treatment with the greatest impact on survival, with a median overall survival of up to 5 years (28, 29).

The treatment of retroperitoneal liposarcomas requires multidisciplinary collaboration. In cases in which complete resection is not feasible or for managing high-grade sarcomas, preoperative radiotherapy and chemotherapy may be considered. However, preoperative radiotherapy is not currently recommended for resectable retroperitoneal liposarcomas (5). The prognosis of patients with unresectable local and/or metastatic dedifferentiated liposarcoma is poor. Anthracycline-based therapy is regarded as the standard initial treatment for advanced dedifferentiated liposarcoma, whereas eribulin is currently approved for the treatment of patients with unresectable or metastatic liposarcoma who have previously received anthracycline-based therapy (30, 31). However, their efficacy is limited. A recent *in vitro* study using human tissues as research subjects has shown that trabectedin, as a second-line chemotherapy drug, has promising efficacy in treating patients with refractory or recurrent liposarcoma by regulating the tumor microenvironment and extracellular matrix (32). However, the efficacy of immunotherapy, radiofrequency ablation, CAR-T cell therapy, and TCR-T cell therapy remains controversial, and these novel treatment approaches are currently in the research stage.

Colonic invasion is a rare manifestation of liposarcomas. A literature review identified only four cases of colonic invasion, and a summary of these cases yielded four hypotheses to explain the pathogenesis of this clinical manifestation: (1) The most obvious mechanism is histological invasion of the colon, extending to the mucosa; (2) ischemic colitis secondary to vascular compression; (3) invasion of a portion of the digestive tract associated with strong adhesion of the tumor to the colon; (4) Long-term exogenous compression of the colon leads to partial invasion of the histological layers and mucosal atrophy (33–36).

## Patient perspective

Since I was diagnosed with a retroperitoneal tumor, it has caused immense psychological stress and severely affected my quality of life. The doctors helped me make the correct diagnosis and completely remove the tumor. My fear and anxiety had significantly improved. Physical and psychological healing was achieved. I think I have been successfully treated.

## Conclusion

Retroperitoneal dedifferentiated liposarcomas with colonic invasion are an extremely rare. Retroperitoneal liposarcomas typically exhibit compressive rather than invasive behavior toward adjacent organs. However, dedifferentiated liposarcomas are characterized by local invasiveness and a higher recurrence rate. The association between dedifferentiated liposarcoma and invasive behavior observed in this patient underscores the importance of multidisciplinary collaboration as the primary treatment strategy. Nevertheless, complete tumor resection remains crucial for improving the prognosis and reducing the high local recurrence rate. Although surgical resection can temporarily address the short-term issues of patients with retroperitoneal liposarcoma, more effective treatment regimens still need to be explored to reduce the risk of recurrence or metastasis and improve patient survival rates.

## Data Availability

The original contributions presented in the study are included in the article/supplementary material. Further inquiries can be directed to the corresponding author.
